# Healthcare-associated infections and antimicrobial resistance in Canadian acute care hospitals, 2016–2020

**DOI:** 10.14745/ccdr.v48i78a03

**Published:** 2022-07-07

**Authors:** Canadian Nosocomial Infection Surveillance Program

**Affiliations:** 1 Centre for Communicable Diseases and Infection Control, Public Health Agency of Canada, Ottawa, ON

**Keywords:** healthcare-associated infections, community-associated infections, antimicrobial resistance, surveillance, *Clostridioides difficile* infection, methicillin-resistant *Staphylococcus aureus*, vancomycin-resistant *Enterococci*, carbapenemase-producing *Enterobacterales*, *Escherichia coli*, Canadian Nosocomial Infection Surveillance Program

## Abstract

**Background:**

Canadians experience increased morbidity, mortality and healthcare costs due to healthcare-associated infections (HAIs) and antimicrobial resistance (AMR). The Canadian Nosocomial Infection Surveillance Program (CNISP) collects and utilizes epidemiologic and laboratory surveillance data to inform infection prevention and control and antimicrobial stewardship programs and policies. The objective of this report is to describe the epidemiologic and laboratory characteristics and trends of HAIs and AMR from 2016 to 2020 using surveillance data provided by Canadian hospitals participating in the CNISP.

**Methods:**

Data were collected from 87 Canadian sentinel acute care hospitals between January 1, 2016, and December 31, 2020, for *Clostridioides difficile* infection (CDI), methicillin-resistant *Staphylococcus aureus* (MRSA) bloodstream infections, vancomycin-resistant *Enterococci* (VRE) bloodstream infections and carbapenemase-producing *Enterobacterales* (CPE). Case counts, rates, outcome data, molecular characterization and antimicrobial resistance profiles are presented.

**Results:**

From 2016 to 2020, increases in rates per 10,000 patient days were observed for MRSA bloodstream infections (33%; 0.84–1.12, *p*=0.037), VRE bloodstream infections (72%; 0.18–0.31, *p*=0.327), and CPE infections (67%, 0.03–0.05, *p*=0.117) and colonizations (86%, 0.14–0.26, *p*=0.050); however, CDI rates decreased by 8.5% between 2016 and 2020 (from 5.77–5.28, *p*=0.050).

**Conclusion:**

Surveillance findings from a national network of Canadian acute care hospitals indicate that rates of MRSA and VRE bloodstream infections, CPE infections and colonizations have increased substantially between 2016 and 2020 while rates of CDI have decreased. The collection of detailed, standardized surveillance data and the consistent application of infection prevention and control practices in acute care hospitals are critical in reducing the burden of HAIs and AMR infections in Canada. Further investigations into the impact of coronavirus disease 2019 and associated public health measures are underway.

## Introduction

Healthcare-associated infections (HAIs), including those caused by antimicrobial resistant organisms (AROs), are an ongoing threat to the health and safety of patients. The morbidity and mortality caused by HAIs place significant burden on patients and healthcare resources ((1–5)). A 2017 Canadian point-prevalence survey estimated that 7.9% of patients had at least one HAI; results comparable to those reported by the European Centre for Disease Prevention and Control where HAI prevalence among tertiary hospitals was estimated to be 7.1% ((6,7)). A similar 2015 point-prevalence study in the United States estimated that there were 687,000 HAIs in acute care hospitals ((8)). During the coronavirus disease 2019 (COVID-19) pandemic that was declared on March 11, 2020 ((9)), changes in hospital infection prevention and control and antimicrobial stewardship efforts may have had impacts on rates of HAIs and AMR ((10)).

Antimicrobial resistance (AMR) has been recognized as a growing danger to global health ((11)). Worldwide, an estimated 700,000 people die of resistant infections each year ((12)). In Canada, it is estimated that 1 in 19 deaths are attributable to resistant bacterial infections. The cost of AMR to the healthcare sector is $1.4 billion per year and is projected to increase to $7.6 billion per year by 2050 ((13)). Global surveillance, improved antibiotic stewardship, enhanced infection prevention and control and public awareness are vital to curbing existing and emerging infections and identifying patterns of antimicrobial resistance.

In Canada, the Public Health Agency of Canada collects national data on various HAIs and AMR through the Canadian Nosocomial Infection Surveillance Program (CNISP). Established in 1994, CNISP is a collaboration between the Public Health Agency of Canada, the Association of Medical Microbiology and Infectious Disease Canada and sentinel hospitals from across Canada. The goal of CNISP is to facilitate and inform the prevention, control and reduction of HAIs and AROs in Canadian acute care hospitals through active surveillance and reporting.

Consistent with the World Health Organization’s core components of infection prevention and control ((10)), CNISP performs consistent, standardized surveillance to reliably estimate HAI burden, establish benchmark rates for national and international comparison, identify potential risk factors and assess and inform specific interventions to improve patient health outcomes. Data provided by CNISP directly supports the collaborative goals outlined in the 2017 Pan-Canadian Framework for Action for tackling antimicrobial resistance and antimicrobial use ((11)).

In this report, we describe the most recent HAI and AMR surveillance data collected from CNISP participating hospitals between 2016 and 2020.

## Methods

### Design

The Canadian Nosocomial Infection Surveillance Program conducts prospective, sentinel surveillance for HAIs (including AROs).

### Case definitions

Standardized case definitions for healthcare-associated (HA) and community-associated (CA) infections were used. Refer to **Annex A** for full case definitions.

### Data sources

Between January 1, 2016, and December 31, 2020, participating hospitals submitted epidemiologic data on cases meeting the respective case definitions for *Clostridioides difficile* infection (CDI), methicillin-resistant *Staphylococcus aureus* bloodstream infections (MRSA BSI), vancomycin-resistant *Enterococci* bloodstream infections (VRE BSI) and carbapenemase-producing *Enterobacterales* (CPE) infections and colonizations. In 2020, 87 hospitals across Canada participated in HAI surveillance and are further described in **Table 1**. In 2020, nearly half of patient admissions captured in CNISP HAI surveillance were from medium-sized adult (sites=21, 27%) and mixed hospitals (sites=14, 22%) (**Supplemental file** **Figure S1**).

**Table 1:** Summary of hospitals participating in the Canadian Nosocomial Infection Surveillance Program, by region, 2020

Details of participating hospitals	Western^a^	Central^b^	Eastern^c^	Northern^d^	Total
Total number of hospitals	28	32	26	1	87
Hospital type					
Adult^e^	12	21	16	0	49
Mixed	12	7	9	1	29
Paediatric	4	4	1	0	9
Hospital size					
Small (1–200 beds)	10	8	18	1	37
Medium (201–499 beds)	11	17	8	0	36
Large (500+ beds)	7	7	0	0	14
Admissions and discharge					
Total number of beds	9,617	12,130	3,302	22	25,071
Total number of admissions	424,296	494,428	133,894	2,271	1,054,889
Total number of patient days	3,137,774	3,721,010	933,042	6,085	7,797,911

^a^ Western refers to British Columbia, Alberta, Saskatchewan and Manitoba

^b^ Central refers to Ontario and Québec

^c^ Eastern refers to Nova Scotia, New Brunswick, Prince Edward Island and Newfoundland and Labrador

^d^ Northern refers to Nunavut

^e^ Seven hospitals classified as “adult” had a neonatal intensive care unit

Epidemiologic (demographic, clinical and outcome data) and denominator data (patient days and patient admissions) were collected and submitted by participating hospitals through the Canadian Network for Public Health Intelligence platform, a secure online data platform.

Reviews of standardized protocols and case definitions were conducted annually by established infectious disease expert working groups and training for data submission was provided as required. Data quality for each surveillance project was periodically evaluated ((14,15)).

### Laboratory data

Patient-linked laboratory isolates (stool samples for CDI cases) were sent to the Public Health Agency of Canada’s National Microbiology Laboratory for molecular characterization and susceptibility testing. The MRSA BSI, VRE BSI, CPE and paediatric CDI isolates were submitted year-round. Adult CDI isolates were submitted annually during a targeted two-month period (March 1 to April 30).

### Statistical analysis

Rates of HAI were calculated and represent infections and/or colonizations identified in patients admitted to CNISP participating hospitals. The HAI rates were calculated by dividing the total number of cases by the total number of patient admissions (multiplied by 1,000) or patient days (multiplied by 10,000). The HAI rates are reported nationally and by region (Western: British Columbia, Alberta, Saskatchewan and Manitoba; Central: Ontario and Québec; Eastern: Nova Scotia, New Brunswick, Prince Edward Island and Newfoundland and Labrador; Northern: Nunavut). Sites that were unable to provide case data were excluded from rate calculations and missing denominator data were estimated, where applicable. Missing epidemiological and molecular data were excluded from analysis. The Mann-Kendall test was used to test trends over time. Significance testing was two-tailed and differences were considered significant at *p*≤0.05.

Where available, attributable and all-cause mortality were reported for HAIs. Attributable mortality rate was defined as the number of deaths per 100 HAI cases where the HAI was the direct cause of death or contributed to death within 30 days after the date of the first positive laboratory or histopathology specimen, as determined by physician review. All-cause mortality rate was defined as the number of deaths per 100 HAI cases 30 days following positive culture.

## Results

### *Clostridioides difficile* infection

Between 2016 and 2020, overall CDI rates significantly decreased by 8.5% (5.77–5.28 infections per 10,000 patient days, *p*=0.050); however, a similar increase of 8.0% in CDI rates (4.89–5.28 per 10,000 patient days) was observed in 2020 compared to 2019 (**Table 2**). Stratified by source of infection, the incidence of HA-CDI significantly decreased by 13.4% from 4.39–3.80 infections per 10,000 patient days (*p*=0.050) (Table S1.1). Community-associated-CDI (Annex A) rates have decreased 3.0% when comparing 2016 to 2020 rates per 1,000 patient admission; however, the decreasing trend was not considered significant (*p*=0.327). Both HA and CA-CDI rates increased in 2020 compared to 2019 (5.0% and 11.1%, respectively). Regionally, HA-CDI rates have steadily decreased across all regions except in the East where rates have remained relatively consistent. For CA-CDI, Eastern and Central region rates have decreased between 2016 and 2020 while Western rates have remained the same. Overall CDI attributable mortality remained low and fluctuated (range: 1.3–2.7 deaths per 100 cases) from 2016 to 2020 (*p*=0.801) (Table 2).

**Table 2:** *Clostridioides difficile* infection data, Canada, 2016–2020^a^

*C. difficile* infection data	Year									
	2016		2017		2018		2019		2020	
Number of infections and incidence rates										
Number of *C. difficile* infection cases	4,008		4,012		3,842		3,595		3,645	
Rate per 1,000 patient admissions	4.34		4.28		4.13		3.71		3.92	
Rate per 10,000 patient days	5.77		5.67		5.39		4.89		5.28	
Number of reporting hospitals	67		68		68		73		82	
Attributable mortality rate per 100 cases (%)^b^	2.4		2.3		1.3		2.3		2.7	
Antimicrobial resistance^c^	n	%	n	%	n	%	n	%	n	%
Clindamycin	145	22.1	149	22.0	307	48.6	219	40.0	66	15.5
Moxifloxacin	103	15.7	114	16.9	70	11.1	64	11.7	28	6.6
Rifampin	9	1.4	14	2.1	10	1.6	5	0.9	4	0.9
Metronidazole	0	0.0	0	0.0	1	0.2	0	0.0	0	0
Total number of isolates tested^d^	657	N/A	676	N/A	632	N/A	547	N/A	426	N/A

Abbreviations: *C. difficile, Clostridioides difficile*; N/A, not applicable

^a^ All *C. difficile* isolates from 2016 to 2020 submitted to National Microbiology Laboratory were susceptible to tigecycline and vancomycin

^b^ Deaths where *C. difficile* infection was the direct cause of death or contributed to death 30 days after the date of the first positive lab specimen or positive histopathology specimen. Mortality data are collected during the two-month period (March and April of each year) for adults (age 18 years and older) and year-round for children (age 1 year to younger than 18 years old). Among paediatric patients, there was no death attributable to healthcare-associated *C. difficile* infection

^c^ *C. difficile* infection isolates are collected for resistance testing during the two-month period (March and April of each year) for adults (age 18 years and older) and year-round for children (age 1 year to younger than 18 years old) from admitted patients only

^d^ Total number reflects the number of isolates tested for each of the antibiotics listed above

The proportion of *C. difficile* isolates resistant to moxifloxacin decreased by 9.1% between 2016 (15.7%, n=103/657) and 2020 (6.6%, n=28/426). Since 2016, moxifloxacin resistance decreased significantly among HA-CDI isolates (11.0%, *p*=0.050) while a smaller non-significant decrease was observed among CA-CDI (3.4%, *p*=0.624) (Table S1.2). All tested *C. difficile* isolates were susceptible to vancomycin and tigecycline. There was a single case of metronidazole resistance in 2018. From 2016 to 2020, the prevalence of ribotype 027 associated with NAP1 decreased for both HA and CA-CDI (5.3% vs. 5.9%, respectively) (Table S1.3).

### Methicillin-resistant *Staphylococcus aureus* bloodstream infections

Between 2016 and 2019, overall MRSA BSI rates significantly increased by 33.3% (0.84–1.12 infections per 10,000 patient days, *p*=0.037), and remained stable in 2020 during the COVID-19 pandemic (**Table 3**). Stratified by case type, a continued steady increase (75%, *p=*0.023) was observed from 2016 to 2020 in CA-MRSA BSI (Annex A) compared to HA-MRSA BSI, which fluctuated over time (Table S2.1). In 2020, HA-MRSA BSI and CA-MRSA BSI rates were highest in Western Canada (0.46 and 0.79 infections per 10,000 patient days, respectively). Among hospital types, HA and CA-MRSA BSI rates have generally remained highest among adult and mixed hospitals. Stratified by hospital size, HA-MRSA BSI rates were highest among large hospitals (500+ beds) since 2018 while CA-MRSA BSI rates have remained highest among medium size hospitals (201–499 beds) since 2019. All-cause mortality decreased 1.7% from 2016 to 2020 (19.1%–17.4%, *p*=0.449) (Table 3). In 2020, all-cause mortality was higher among those with HA-MRSA (19.9%) compared to those with CA-MRSA (15.9%) (data not shown).

**Table 3:** Methicillin-resistant *Staphylococcus aureus* bloodstream infections data, Canada, 2016–2020

MRSA BSI data	Year									
	2016		2017		2018		2019		2020	
Number of infections and incidence rates										
Number of MRSA bloodstream infections	604		606		767		881		845	
Rate per 1,000 patient admissions	0.61		0.61		0.78		0.84		0.83	
Rate per 10,000 patient days	0.84		0.84		1.05		1.12		1.12	
Number of reporting hospitals	64		65		62		69		80	
All-cause mortality rate^a^										
Number of deaths	111		99		144		144		146	
All-cause mortality rate per 100 cases	19.1		16.4		18.8		16.4		17.4	
Antimicrobial resistance^b^	n	%	n	%	n	%	n	%	n	%
Erythromycin	418	78.7	455	81.0	531	75.6	511	75.6	447	72.3
Ciprofloxacin	411	77.4	432	76.9	504	71.8	473	70.0	404	65.4
Clindamycin	230	43.3	239	42.5	290	41.3	144	21.3	202	32.7
Tetracycline	31	5.8	35	6.2	50	7.1	48	7.1	39	6.3
Trimethoprim/sulfamethoxazole	11	2.1	8	1.4	14	2.0	10	1.5	14	2.3
Rifampin	10	1.9	9	1.6	6	0.9	7	1.0	6	1.0
Tigecycline	0	0.0	0	0.0	0	0.0	0	0.0	0	0.0
Daptomycin	5	0.9	5	0.9	0	0.0	0	0.0	4	0.6
Total number of isolates tested^c,d^	531	N/A	562	N/A	702	N/A	676	N/A	618	N/A

Abbreviations: MRSA, methicillin-resistant *S. aureus*; MRSA BSI, methicillin-resistant *S. aureus* bloodstream infection; N/A, not applicable

^a^ Based on the number of cases with associated 30-day outcome data

^b^ All MRSA isolates from 2016 to 2020 submitted to National Microbiology Laboratory were susceptible to linezolid and vancomycin

^c^ In some years, the number of isolates tested for resistance varied by antibiotic

^d^ Total number reflects the number of isolates tested for each of the antibiotics listed above

Clindamycin resistance among MRSA isolates decreased by 10.6% between 2016 (43.3%, n=230/531) and 2020 (32.7%, n=202/618) (Table 3). Since 2016, the proportion of MRSA isolates with erythromycin and ciprofloxacin resistance has decreased, yet remains high (72.3% and 65.4% in 2020, respectively). Between 2016 and 2020, daptomycin resistance was detected in 14 isolates. All tested MRSA isolates from 2016 to 2020 were susceptible to linezolid and vancomycin.

Stratified by case type, clindamycin resistance among HA-MRSA isolates (45.8%) was, on average, consistently higher from 2016 to 2020 compared to CA-MRSA isolates (34.1%) during the same period (Table S2.2). There were no other notable differences in antibiotic resistance patterns by MRSA BSI case type.

Between 2016 and 2020, the proportion of epidemic types identified as CMRSA2 (USA100/800) and most commonly associated with MRSA infections acquired in a hospital or healthcare setting continued to decrease; from 33.6% of all isolates in 2016 to 21.2% in 2020. The proportion of epidemic types identified as CMRSA7 (USA400) and CMRSA10 (USA300) and most commonly associated with MRSA infections acquired in the community continued to increase and account for the largest proportion of all isolates from 2016 (52.8%) to 2020 (63.8%). The CMRSA10 (USA300) was the most common epidemic type identified from 2016 to 2020, with 50.2% identified in 2020 (n=311/620) (Table S2.3).

### Vancomycin-resistant *Enterococci* bloodstream infections

From 2016 to 2020, VRE BSI rates increased 72.2%, from 0.18 to 0.31 infections per 10,000 patient days, with the highest rate of 0.35 infections per 10,000 patient days observed in 2018 (**Table 4**). During the COVID-19 pandemic in 2020, VRE BSI rates in the CNISP network remained stable compared to 2019. Regionally, VRE BSI rates were highest in Western and Central Canada (0.36 and 0.33 infections per 10,000 patient days in 2019, respectively) with few VRE BSIs reported in Eastern Canada (range: 0–0.03 infections per 10,000 patient days) (Table S3.1). In 2020 compared to 2019, VRE BSI rates decreased among large (500+ beds) and small (1–200 beds) hospitals while increasing by 28.6% (0.28–0.36 infections per 10,000 patient days) among medium (201–499 beds) hospitals.

**Table 4:** Vancomycin-resistant *Enterococci* bloodstream infections data, Canada, 2016–2020

VRE BSI data	Year									
	2016		2017		2018		2019		2020	
Vancomycin-resistant *Enterococci* bloodstream infections data										
Number of VRE BSI infections	121		154		246		247		207	
Rate per 1,000 patient admissions	0.13		0.16		0.26		0.23		0.24	
Rate per 10,000 patient days	0.18		0.23		0.35		0.32		0.31	
Number of reporting hospitals	59		59		59		68		62	
Antimicrobial resistance of *Enterococcus faecium* isolates	n	%	n	%	n	%	n	%	n	%
Ampicillin	91	100	116	100	181	100	169	100	112	97.4
Chloramphenicol	2	2.2	11	9.5	4	2.2	28	16.6	22	19.1
Ciprofloxacin	91	100	116	100	181	100	169	100	113	98.3
Daptomycin^a^	7	7.7	10	8.6	12	6.6	7	4.1	4	3.5
Erythromycin	83	91.2	108	93.1	173	95.6	162	95.9	108	93.9
High-level gentamicin	12	13.2	45	38.8	77	42.5	56	33.1	30	26.1
Levofloxacin	91	100	116	100	179	98.9	169	100	112	97.4
Linezolid	1	1.1	0	0	2	1.1	3	1.8	0	0
Nitrofurantoin	35	38.5	52	44.8	55	30.4	68	40.2	40	34.8
Penicillin	91	100	116	100	181	100.0	169	100	113	98.3
Quinupristin/Dalfopristin	9	9.9	8	6.9	18	9.9	18	10.7	8	7.0
Rifampicin	85	93.4	110	94.8	163	90.1	155	91.7	98	85.2
High-level streptomycin	32	35.2	39	33.6	60	33.1	43	25.4	23	20.0
Tetracycline	46	50.5	66	56.9	108	59.7	119	70.4	72	62.6
Tigecycline	0	0	0	0	1	0.6	0	0	0	0
Vancomycin	88	96.7	111	95.7	176	97.2	166	98.2	110	95.7
Total number of isolates tested^b^	91	N/A	116	N/A	181	N/A	169	N/A	115	N/A

Abbreviations: VRE BSI, vancomycin-resistant *Enterococci* bloodstream infection; N/A, not applicable

^a^ Daptomycin does not have intermediate or resistant breakpoints in 2016, 2017 & 2018. Clinical and Laboratory Standards Institute (CLSI) resistance breakpoints came into effect in 2019

^b^ Total number reflects the number of isolates tested for each of the antibiotics listed above

Note: Aggregate mortality data reported in-text due to fluctuations in the small numbers of VRE BSI deaths reported each year

Vancomycin-resistant *Enterococci* bloodstream infections were predominantly healthcare-associated, as 93.2% (n=887/952) reported from 2016 to 2020 were acquired in a healthcare facility (Table S3.2). All-cause mortality remained high (32.7%) from 2016 to 2020.

Between 2016 and 2020, high-level gentamycin resistance among VRE BSI isolates (*Enterococcus faecium*) increased from 13.2% to 26.1%; however, a 7.0% decrease was observed more recently between 2019 and 2020. Daptomycin non-susceptibility was first identified in 2016 (n=7/91, 7.7%) and decreased to 3.5% (n=4/115) in 2020 (Table 4). Since 2016, the majority (98.4%–100%) of VRE BSI isolates were identified as *Enterococcus faecium*; however, in 2018, three *E. faecalis* VRE BSI isolates were identified (Table S3.3). Among *E. faecium* isolates, the proportion identified as sequence type 1478 was highest in 2018 (38.7%, n=70/181) and decreased in 2020 (17.6%, n=21/119; *p*<0.001) (Table S3.4).

### Carbapenemase-producing *Enterobacterales*

From 2016 to 2020, CPE infection rates have remained low but increased from 0.03 to 0.05 infections per 10,000 patient days (*p*=0.117), while a significant increase (85.7%) was observed in CPE colonization rates (from 0.14 to 0.26 colonizations per 10,000 patient days, *p*=0.050) (**Table 5**). Both CPE infections and colonizations rates decreased in 2020 compared to 2019 (16.7% and 10.3%, respectively).

**Table 5:** Carbapenemase-producing *Enterobacterales* data, Canada, 2016–2020^a^

CPE data	Year										
	2016		2017			2018		2019		2020	
Number of infections and incidence rates											
Number of CPE infections	21		20			36		48		35	
Infection rate per 1,000 patient admissions	0.02		0.02			0.04		0.05		0.04	
Infection rate per 10,000 patient days	0.03		0.03			0.05		0.06		0.05	
Number of CPE colonizations	88		112			142		214		190	
Colonization rate per 1,000 patient admissions	0.10		0.12			0.16		0.21		0.20	
Colonization rate per 10,000 patient days	0.14		0.18			0.22		0.29		0.26	
Number of reporting hospitals	55		56			57		64		72	
Drugs tested for antimicrobial resistance											
Antibiotics^b,c^	n	%		n	%	n	%	n	%	n	%
Piperacillin-Tazobactam	116	72.0		159	85.0	210	92.1	237	90.8	184	87.6
Ceftriaxone	149	92.5		173	92.5	212	93.0	250	95.8	186	88.6
Ceftazidime	139	86.3		160	85.6	192	84.2	233	89.3	173	82.4
Meropenem	140	87.0		159	85.0	198	86.8	190	72.8	130	61.9
Ciprofloxacin	133	82.6		138	73.8	158	69.3	183	70.1	150	71.4
Amikacin	42	26.1		32	17.1	44	19.3	23	8.8	16	7.6
Gentamicin	62	38.5		64	34.2	80	35.1	86	33.0	61	29.1
Tobramycin	75	46.6		71	38.0	101	44.3	121	46.4	78	37.1
Trimethoprim-sulfamethoxazole	102	63.4		113	60.4	143	62.7	193	73.9	160	76.2
Tigecycline	32	19.9		18	9.6	30	13.2	36	13.8	0	0
Total number of isolates tested^d^	161	N/A		187	N/A	228	N/A	261	N/A	210	N/A
Carbapenemases identified											
KPC	84	52.2		86	46.0	122	53.5	127	48.7	82	39.1
NDM	45	28.0		53	28.3	59	25.9	74	28.4	66	31.4
OXA-48	20	12.4		33	17.6	30	13.2	40	15.3	45	21.4
SME^e^	4	2.5		2	1.1	4	1.8	1	0.4	2	1
NDM/OXA-48	4	2.5		5	2.7	6	2.6	10	3.8	7	3.3
GES	1	0.6		1	0.5	1	0.4	2	0.8	0	0
IMP	0	0.0		0	0.0	3	1.3	1	0.4	1	0.5
NMC	2	1.2		4	2.1	2	0.9	4	1.5	6	2.9
VIM	2	1.2		3	1.6	3	1.3	3	1.1	0	0
Other	0	0.0		0	0.0	0	0.0	0	0.0	0	0
Total number of isolates tested^f^	161	N/A		187	N/A	228	N/A	261	N/A	210	N/A

Abbreviations: CPE, carbapenemase-producing *Enterobacterales*; GES, Guiana extended-spectrum β-lactamase; IMP, active-on-imipenem; KPC, *Klebsiella pneumoniae* carbapenemase; NDM, New Delhi metallo-β-lactamase; NMC, not metalloenzyme carbapenemase; OXA-48, Oxacillinase-48; N/A, not applicable; SME, *Serratia marcescens* enzymes; VIM, Verona integron-encoded metallo-β-lactamase

^a^ Includes data for all CPE isolates submitted

^b^ All isolates were resistant to ampicillin, and all but one to cefazolin. All carbapenemase-producing organism isolates were screened for the *mcr*-type gene which is an acquired gene associated with colistin resistance

^c^ The denominator for some drugs were adjusted as minimum inhibitory concentration values were not given in all cases due to VITEK^®^ algorithms

^d^ Total number reflects the number of isolates tested for each of the antibiotics listed above

^e^ Only found in *Serratia marcescens*

^f^ Some isolates contain multiple carbapenemases therefore the total number of isolates tested and the number of carbapenemases indicated may not match

Note: Aggregate mortality data reported in-text due to fluctuations in the small numbers of CPE deaths reported each year

From 2016 to 2020, the majority of CPE infections (97.5%) were identified in Central (50.0%, n=80/160) and Western Canada (47.5%, n=76/160) while few infections were identified in the East (2.5%; n=4/160) (Table S4.1). During this same period, most CPE colonizations were identified in Central Canada (80.4%; n=600/746), followed by Western Canada (19.1%, n=143/746), while only three colonizations were reported in Eastern Canada (Table S4.2). From 2016 to 2020, large hospitals (500+ beds) reported the highest rates of CPE infections (0.04–0.09 infections per 10,000 patient days); however, small hospitals (1–200 beds) reported the highest CPE infection rates in 2019 (0.10 infections per 10,000 patient days). The CPE colonization rates remained highest among large hospitals from 2016 to 2020 (range: 0.25–0.35 infections per 10,000 patient days).

Thirty day all-cause mortality was 15.2% (n=22/145) among CPE-infected patients. Among all CPE cases reported from 2016 to 2020, 39.2% (n=312/795) reported travel outside of Canada and of those, 83.3% (n=240/288) received medical care while abroad.

From 2016 to 2020, the prevalence of amikacin and gentamicin resistance among CPE isolates decreased by 18.5% and 9.4%, respectively, while trimethoprim-sulfamethoxazole resistance increased by 12.8% (Table 5). The predominant carbapenemases identified in Canada were *Klebsiella pneumoniae* carbapenemase (KPC), New Delhi metallo-β-lactamase (NDM), and Oxacillinase-48 (OXA-48), accounting for 91.9% of identified carbapenemases in 2020.

Among submitted isolates from 2016 to 2020, the proportion of carbapenemase-producing pathogens identified as *Escherichia coli* increased 11.9% while those identified as *K. pneumoniae* and *Acinetobacter baumannii* decreased by 10.9% each (Table S5).

## Discussion

Surveillance data collected via CNISP have shown that between 2016 and 2020 infection rates (including both HA and CA-cases) in Canada have decreased 8.5% for CDI, but increased for MRSA BSI and VRE BSI (33.3% and 72.2%, respectively). The CPE infection rates increased, but remained low; however, colonizations increased 85.7%. The COVID-19 pandemic has potentially had mixed impacts on the rates of HAIs in Canada and in the United States ((16)). Further investigation is required to assess the influence of pandemic-related factors that may be attributed to the changes in observed rates of HAIs, such as public health measures implemented in both the hospital and the community, population travel and mobility, changes in infection control practices, screening, laboratory testing and antimicrobial stewardship ((10)).

The CDI rates in Canada declined and followed similar trends observed globally; however, rates remained higher in North America relative to other regions ((17)). In Canada, rates of CDI during the 2020 COVID-19 pandemic were higher than those observed in 2019 and contrast with results seen in the United States where CDI rates have continued to decline ((16)).

The CDI moxifloxacin resistance decreased in Canada to 6.6% in 2020 and remained lower than previously published weighted pooled resistance data for North America (44.0%) and Asia (33.0%) and corresponds to the declining prevalence of ribotype 027 ((18,19)). The overall reduction in CDI rates across Canada suggests improvements in infection prevention and control practices and quality-improvement initiatives such as hand hygiene compliance, environmental cleaning, improved diagnostic techniques and antibiotic stewardship ((20,21)). The decline of RT027 from 2016 to 2020 may also have influenced the decline in CDI rates among CNISP hospitals as this ribotype has been associated with increased virulence and fluoroquinoline resistance ((22)).

The rise in MRSA BSI rates in Canada, attributed to the increase in CA-MRSA BSI rates, is concerning due to the severe clinical outcomes, increased length of hospital stays and increased healthcare costs associated with BSI’s among admitted patients ((23–26)). A reduction in clindamycin resistance from 2016 to 2019 is most likely associated with the decrease in the proportion of CMRSA2 epidemic type identified among tested isolates ((27)). Compared to the increase observed in MRSA BSI rates in Canada, MRSA BSI rates in select large Australian tertiary care hospitals were lower and fluctuated between 2016 and 2019 ((28)). Similarly, in England, a plateau in MRSA BSI rates has been observed since 2015 (1.4–1.5 per 100,000 population and 0.8–0.9 hospital-onset cases per 100,000 bed days) ((29)). Both globally and in Canada, the prevalence of CA-MRSA is increasing and may provide a reservoir that could contribute to the increasing number of patients identified with CA-MRSA admitted to hospitals ((30,31)). The increasing rate of patients hospitalized with MRSA BSI acquired in the community observed in CNISP data suggests that further strategies to reduce or prevent MRSA infections in the community may be needed. Although beyond the scope of CNISP, studies at the broader population level to identify the prevalence of MRSA in the community, especially among populations at increased risk of contracting CA-MRSA, such as children, athletes, incarcerated populations, people who live in crowded conditions or people who inject drugs, may be worthwhile and could help to inform prevention strategies in the community ((32)).

The increasing rates of VRE BSI in Canadian acute care hospitals are of concern as this infection is associated with a high mortality and increased hospital burden ((33–35)). The increase in VRE BSI rates observed among CNISP hospitals may be linked to changes in infection control policies, specifically the discontinuation of VRE screening and isolation programs in some Canadian acute care hospitals ((36)). Additionally, the rise in VRE BSI rates from 2013 to 2018 and subsequent decrease in 2019 and 2020 coincides with the emergence and decline of the *pstS*-null sequence type 1478 (ST1478) ((37)). The ST1478 sequence type is associated with daptomycin non-susceptibility and high-level gentamicin resistance, and the resistance patterns among VRE BSI isolates for these two antibiotics correspond to the trend in ST1478. It is important to note that the observed VRE BSI trends are, for the most part, being driven by a limited number of hospitals that have experienced outbreaks while caring for high risk patients (e.g. bone marrow transplants, solid organ transplants, cancer patients, etc.) ((38)). Similarly, increasing trends in prevalence of VRE BSI have also been observed in Europe ((39–42)), which may be associated, in part, with the introduction and spread of a new clone and gaps in infection prevention practices ((37,41)).

The CPE infections are of clinical significance and public health concern as they are associated with significant morbidity and mortality, limited treatment options and an ability to spread rapidly in healthcare settings ((43–47)). The incidence of CPE infection in Canada remains low; however, an 85.7% increase in CPE colonization rates was observed over the same period of time. Recent decreases in CPE infection and colonization rates in 2020 require further research to investigate the impact of changes in previously identified risk factors such as travel and receipt of healthcare in high-risk areas, as well as changes to infection control practices such as patient screening ((44,48–50)).

Data on the incidence of CPE in other countries remains limited ((51)); however, a few countries have also reported a low but increasing incidence of CPE ((52,53)). Increased awareness and changes in screening and testing practices may reflect the increase in CPE colonization. Coordinated public health action, including strict implementation of infection control measures such as enquiry regarding travel, and enhanced surveillance are essential in reducing the transmission of CPE in Canadian acute care hospitals.

### Strengths and limitations

The CNISP collects standardized and detailed epidemiological and laboratory-linked data from 87 sentinel hospitals across Canada to provide national HAI and AMR trends that can be used for benchmarking hospital infection prevention and control practices in serving to reduce HAIs and AROs in Canadian acute care hospitals. It is important to note that data included in this report include the COVID-19 pandemic, and 2020 rates of HAI’s and AMR may be impacted by changes in hospital admissions, mobility and national, regional, local and hospital-based infection prevention and control measures.

The epidemiologic data collected by CNISP were limited to the information available in patient charts. Turnover of hospital staff reviewing medical charts may affect the consistent application of CNISP definitions and data quality over time; however, these data are collected by experienced and training infection prevention and control staff who receive periodic training with respect to CNISP methods and definitions. Data quality assessments are also conducted to maintain and improve data quality. The CNISP network may not fully represent the general inpatient population in Canada; however, efforts in recruitment have increased representation and coverage of Canadian acute care beds from 27% to 30% from 2016 to 2020, particularly among Northern, rural communities and Indigenous populations.

### Next steps

Continued recruitment of Canadian acute care hospitals to increase acute care bed coverage from all ten provinces and three territories is ongoing in order to improve the quality and representativeness of Canadian HAI estimates. Furthermore, an enhanced hospital screening practice survey is conducted annually to better understand changes in HAI rates across Canada. In recent years, CNISP has initiated surveillance for new and emerging pathogens, such as *Candida auris,* and epidemiologic and laboratory-led working groups were formed to further investigate new pathogens such as VRE BSI ST1478 and extensively drug-resistant CPE. In 2019, CNISP re-established viral respiratory infection surveillance to collect and report detailed epidemiologic information on patients hospitalized with viral respiratory infections. This surveillance was expanded in 2020 to include patients hospitalized with COVID-19. The CNISP continues to support the national public health response to the COVID-19 pandemic. Future studies aim to analyze the impact of the COVID-19 pandemic on HAI rates and AMR.

## Conclusion

Findings from surveillance conducted by a national network of Canadian acute care hospitals indicate that rates of MRSA BSI, VRE BSI and CPE infections and colonizations substantially increased between 2016 and 2020 while rates of CDI decreased. Ongoing surveillance and reporting of epidemiologic and laboratory data are essential to inform infection prevention and control and antimicrobial stewardship policies to help reduce the burden of HAI and impact of AMR in Canadian acute care hospitals.

## Annex A: Surveillance case definitions and eligibility criteria, 2020

### *Clostridioides difficile* infection

A “primary” episode of *Clostridioides difficile* infection (CDI) is defined either as the first episode of CDI ever experienced by the patient or a new episode of CDI that occurs greater than eight weeks after the diagnosis of a previous episode in the same patient.

A patient is identified as having CDI if:

- The patient has diarrhea or fever, abdominal pain and/or ileus AND a laboratory confirmation of a positive toxin assay or positive polymerase chain reaction (PCR) for *C. difficile* (without reasonable evidence of another cause of diarrhea)

OR

- The patient has a diagnosis of pseudomembranes on sigmoidoscopy or colonoscopy (or after colectomy) or histological/pathological diagnosis of CDI

OR

- The patient is diagnosed with toxic megacolon (in adult patients only)

#### Diarrhea is defined as one of the following:

- More watery/unformed stools in a 36-hour period

OR

- More watery/unformed stools in a 24-hour period and this is new or unusual for the patient (in adult patients only)

#### Exclusion:

- Any patients younger than one year
- Any paediatric patients (aged one year to younger than 18 years) with alternate cause of diarrhea found (i.e. rotavirus, norovirus, enema or medication, etc.) are excluded even if *C. difficile* diagnostic test result is positive

#### CDI case classification

Once a patient has been identified with CDI, the infection will be classified further based on the following criteria and the best clinical judgment of the healthcare and/or infection prevention and control practitioner.

#### Healthcare-associated (acquired in your facility) CDI case definition

- Related to the current hospitalization:
  - The patient’s CDI symptoms occur in your healthcare facility three or more days (or 72 hours or longer) after admission
- Related to a previous hospitalization:
  - Inpatient: the patient’s CDI symptoms occur less than three days after the current admission (or fewer than 72 hours) AND the patient had been previously hospitalized at your healthcare facility and discharged within the previous four weeks
  - Outpatient: the patient presents with CDI symptoms at your emergency room (ER) or outpatient location AND the patient had been previously hospitalized at your healthcare facility and discharged within the previous four weeks
- Related to a previous healthcare exposure at your facility:
  - Inpatient: the patient’s CDI symptoms occur less than three days after the current admission (or fewer than 72 hours) AND the patient had a previous healthcare exposure at your facility within the previous four weeks
  - Outpatient: the patient presents with CDI symptoms at your ER or outpatient location AND the patient had a previous healthcare exposure at your facility within the previous four weeks

#### Healthcare-associated (acquired in any other healthcare facility) CDI case definition

- Related to a previous hospitalization at any other healthcare facility:
  - Inpatient: the patient’s CDI symptoms occur less than three days after the current admission (or fewer than 72 hours) AND the patient is known to have been previously hospitalized at any other healthcare facility and discharged/transferred within the previous four weeks
  - Outpatient: the patient presents with of CDI symptoms at your ER or outpatient location AND the patient is known to have been previously hospitalized at any other healthcare facility and discharged/transferred within the previous four weeks
- Related to a previous healthcare exposure at any other healthcare facility
  - Inpatient: the patient’s CDI symptoms occur less than three days after the current admission (or fewer than 72 hours) AND the patient is known to have a previous healthcare exposure at any other healthcare facility within the previous four weeks
  - Outpatient: the patient presents with CDI symptoms at your ER or outpatient location AND the patient is known to have a previous healthcare exposure at any other healthcare facility within the previous four weeks

#### Healthcare-associated CDI but unable to determine which facility

The patient with CDI DOES meet both definitions of healthcare-associated (acquired in your facility) and healthcare-associated (acquired in any other healthcare facility), but unable to determine to which facility the case is primarily attributable to.

#### Community-associated CDI case definition

- Inpatient: the patient’s CDI symptoms occur less than three days (or fewer than 72 hours) after admission, with no history of hospitalization or any other healthcare exposure within the previous 12 weeks
- Outpatient: the patient presents with CDI symptoms at your ER or outpatient location with no history of hospitalization or any other healthcare exposure within the previous 12 weeks

#### Indeterminate CDI case definition

The patient with CDI does NOT meet any of the definitions listed above for healthcare-associated or community-associated CDI. The symptom onset was more than four weeks but fewer than 12 weeks after the patient was discharged from any healthcare facility or after the patient had any other healthcare exposure.

### Methicillin-resistant *Staphylococcus aureus* (MRSA) infection

#### MRSA bloodstream infection (BSI) case definition:

- Isolation of *Staphylococcus aureus* from blood

AND

- Patient must be admitted to the hospital

AND

- Is a "newly identified *S. aureus* infection" at a Canadian Nosocomial Infection Surveillance Program (CNISP) hospital at the time of hospital admission or identified during hospitalization.

#### Infection inclusion criteria

- Methicillin-susceptible *Staphylococcus aureus* (MSSA) or MRSA BSIs identified for the first time during this current hospital admission
- MSSA or MRSA BSIs that have already been identified at your site or another CNISP site but are **new** infections

#### Criteria to determine NEW MSSA or MRSA BSI

- Once the patient has been identified with a MSSA or MRSA BSI, they will be classified as a new MSSA or MRSA if they meet the following criteria: more than 14 days since previously treated MSSA or MRSA BSI and in the judgment of infection control physicians and practitioners represents a new infection

#### Infection exclusion criteria

- Emergency, clinic, or other outpatient cases who are **NOT admitted** to the hospital

#### Healthcare-associated (HA) case definition:

Healthcare-associated is defined as an inpatient who meets the following criteria and in accordance with the best clinical judgment of the healthcare and/or infection prevention and control practitioner:

- Patient is on or beyond calendar day 3 of their hospitalization (calendar day 1 is the day of hospital admission)

OR

- Has been hospitalized in your facility in the last 7 days or up to 90 days depending on the source of the infection

OR

- Has had a healthcare exposure at your facility that would have resulted in this bacteremia (using best clinical judgment)

OR

- Any patient who has a bacteremia not acquired at your facility that is thought to be associated with any other healthcare exposure (e.g. another acute-care facility, long-term care, rehabilitation facility, clinic or exposure to a medical device)

#### Healthcare-associated (HA) case definition (newborn):

- The newborn is on or beyond calendar day 3 of their hospitalization (calendar day 1 is the day of hospital admission)
- The mother was **NOT** known to have MRSA on admission and there is no epidemiological reason to suspect that the mother was colonized prior to admission, even if the newborn is fewer than 48 hours of age
- In the case of a newborn transferred from another institution, MSSA or MRSA BSI may be classified as HA your acute-care facility if the organism was **NOT** known to be present and there is no epidemiological reason to suspect that acquisition occurred prior to transfer

#### Community-associated case definition:

- No exposure to healthcare that would have resulted in this bacteremia (using best clinical judgment) and does not meet the criteria for a healthcare-associated BSI

### Vancomycin-resistant *Enterococci* (VRE) infection

#### VRE BSI case definition:

- Isolation of *Enterococcus faecalis* or *faecium* from blood

AND

- Vancomycin MIC **at least** 8 µg/ml

AND

- Patient must be admitted to the hospital

AND

- Is a "newly” identified VRE BSI at a CNISP facility at the time of hospital admission or identified during hospitalization

**A newly identified VRE BSI** is defined as a positive VRE blood isolate more than 14 days after completion of therapy for a previous infection and felt to be unrelated to previous infection in accordance with best clinical judgment by Infection Control physicians and practitioners.

#### Exclusion criteria:

- Emergency, clinic, or other outpatient cases who are **not admitted** to the hospital

#### Healthcare-associated (HA) case definition:

Healthcare-associated is defined as an inpatient who meets the following criteria and in accordance with the best clinical judgment of the healthcare and/or infection prevention and control practitioner:

- Patient is on or beyond calendar day 3 of their hospitalization (calendar day 1 is the day of hospital admission)

OR

- Has been hospitalized in your facility in the last 7 days or up to 90 days depending on the source of the infection

OR

- Has had a healthcare exposure at your facility that would have resulted in this bacteremia (using best clinical judgment)

OR

- Any patient who has a bacteremia not acquired at your facility that is thought to be associated with any other healthcare exposure (e.g. another acute-care facility, long-term care, rehabilitation facility, clinic or exposure to a medical device)

### Carbapenemase-producing *Enterobacterales* (CPE) infection

#### Case eligibility:

- Patient is admitted to a CNISP hospital or presents to a CNISP hospital emergency department or a CNISP hospital-based outpatient clinic
- Laboratory confirmation of carbapenem resistance or carbapenemase production in *Enterobacterales* spp.

Following molecular testing, only isolates determined to be harbouring a carbapenemase are included in surveillance. If multiple isolates are submitted for the same patient in the same surveillance year, only the isolate from the most invasive site is included in epidemiological results (e.g. rates and outcome data). However, antimicrobial susceptibility testing results represent all CPE isolates (including clinical and screening isolates from inpatients and outpatients) submitted between 2016 and 2020; duplicates (i.e. isolates from the same patient where the organism and the carbapenemase were the same) were excluded.

## Annex B: List of supplementary figure and tables

These documents can be accessed on the Supplemental material file.

Figure S1: Number and proportion of patient admissions included in the Canadian Nosocomial Infection Surveillance Program by hospital type and size, 2020

Table S1.1: Cases and incidence rates of healthcare-associated and community-associated *Clostridioides difficile* infection by region, hospital type and hospital size, Canada, 2016–2020

Table S1.2: Antimicrobial resistance of healthcare-associated and community-associated *Clostridioides difficile* infection isolates, Canada, 2016–2020

Table S1.3: Number and proportion of common ribotypes of healthcare-associated and community-associated *Clostridioides difficile* infection cases, Canada, 2016–2020

Table S2.1: Cases and incidence rates of healthcare-associated and community-associated methicillin-resistant *Staphylococcus aureus* bloodstream infections by region, hospital type and hospital size, 2016–2020

Table S2.2: Antimicrobial resistance of healthcare-associated and community-associated methicillin-resistant *Staphylococcus aureus* bloodstream infection isolates, Canada, 2016–2020

Table S2.3: Number and proportion of select methicillin-resistant *Staphylococcus aureus* epidemic types identified

Table S3.1: Number of vancomycin-resistant *Enterococci* bloodstream infections incidence rates by region, hospital type and hospital size, 2016–2020

Table S3.2: Number of healthcare-associated vancomycin-resistant *Enterococci* bloodstream infections and incidence rates by region, hospital type and hospital size, 2016–2020

Table S3.3: Number and proportion of vancomycin-resistant *Enterococci* bloodstream infections isolate types identified, 2016–2020

Table S3.4: Distribution of vancomycin-resistant *Enterococci* bloodstream (*Enterococcus faecium*) sequence type, 2016–2020

Table S4.1: Number of carbapenemase-producing *Enterobacterales* infections and incidence rates by region, hospital type and hospital size, 2016–2020

Table S4.2: Number of carbapenemase-producing *Enterobacterales* colonizations and incidence rates by region, hospital type and hospital size, 2016–2020

Table S5: Number and proportion of main carbapenemase-producing pathogens identified
